# Supplementary material for: Electroacupuncture alleviates pain-like behaviors through modulating DNMT3a/MOR signaling pathway in CCI rats
Source: PLoS One. 2025 Oct 16;20(10):e0334695. doi: 10.1371/journal.pone.0334695 (PMC12530600; doi:10.1371/journal.pone.0334695)
Supplement: S2 Data — (ZIP) [file pone.0334695.s002.zip › Western blot raw data.pdf]

Fig.2D

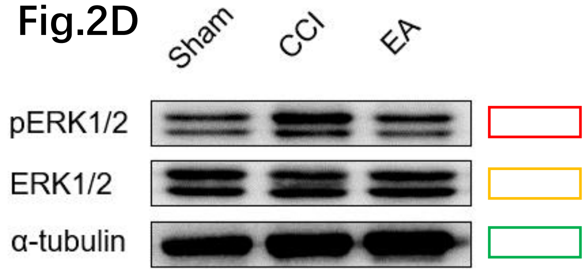

Mini-PROTEAN® Comb, 10-well, 1.5 mm
catalog number:1653365

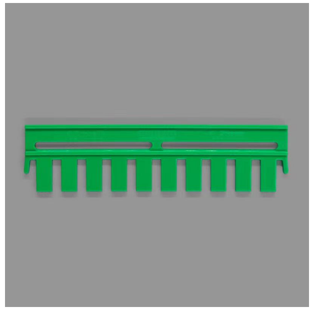

pERK1/2
42/44kd

Tubulin
55kd

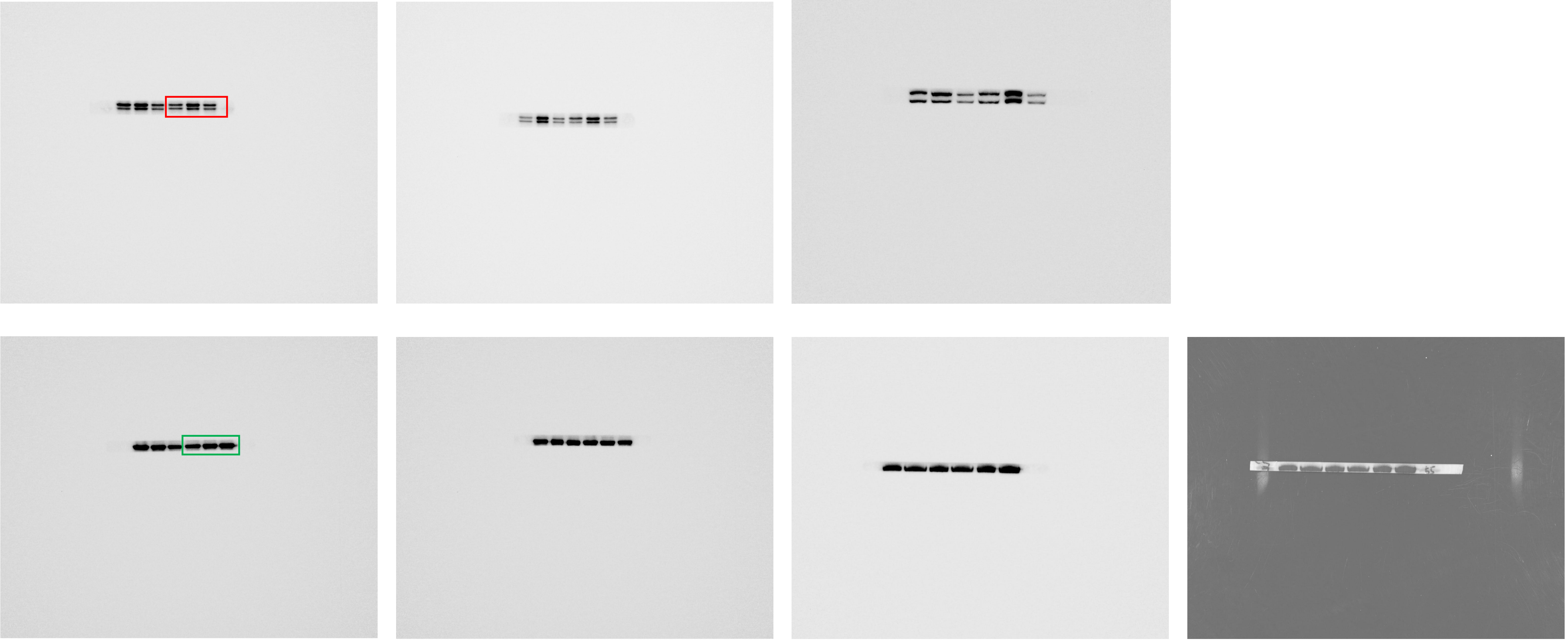

Fig.2D

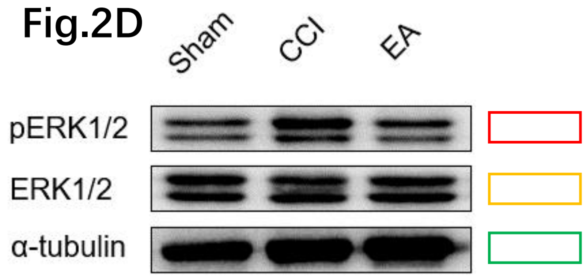

Mini-PROTEAN® Comb, 10-well, 1.5 mm
catalog number:1653365

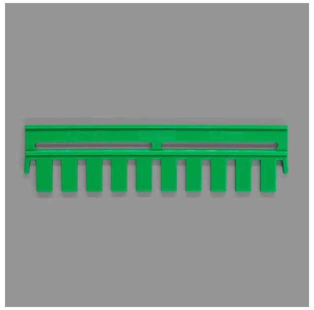

ERK1/2
42/44kd

Tubulin
55kd

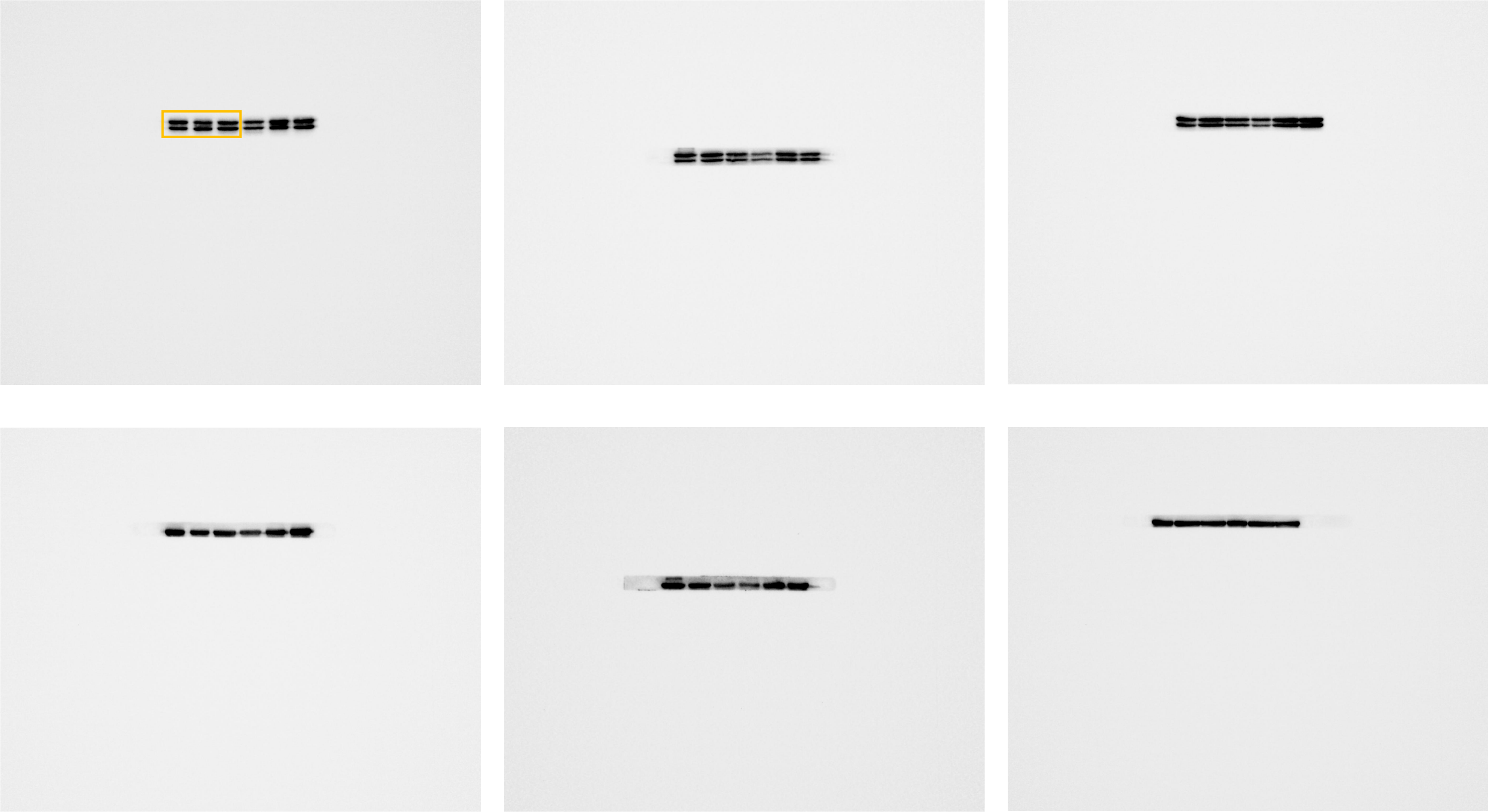

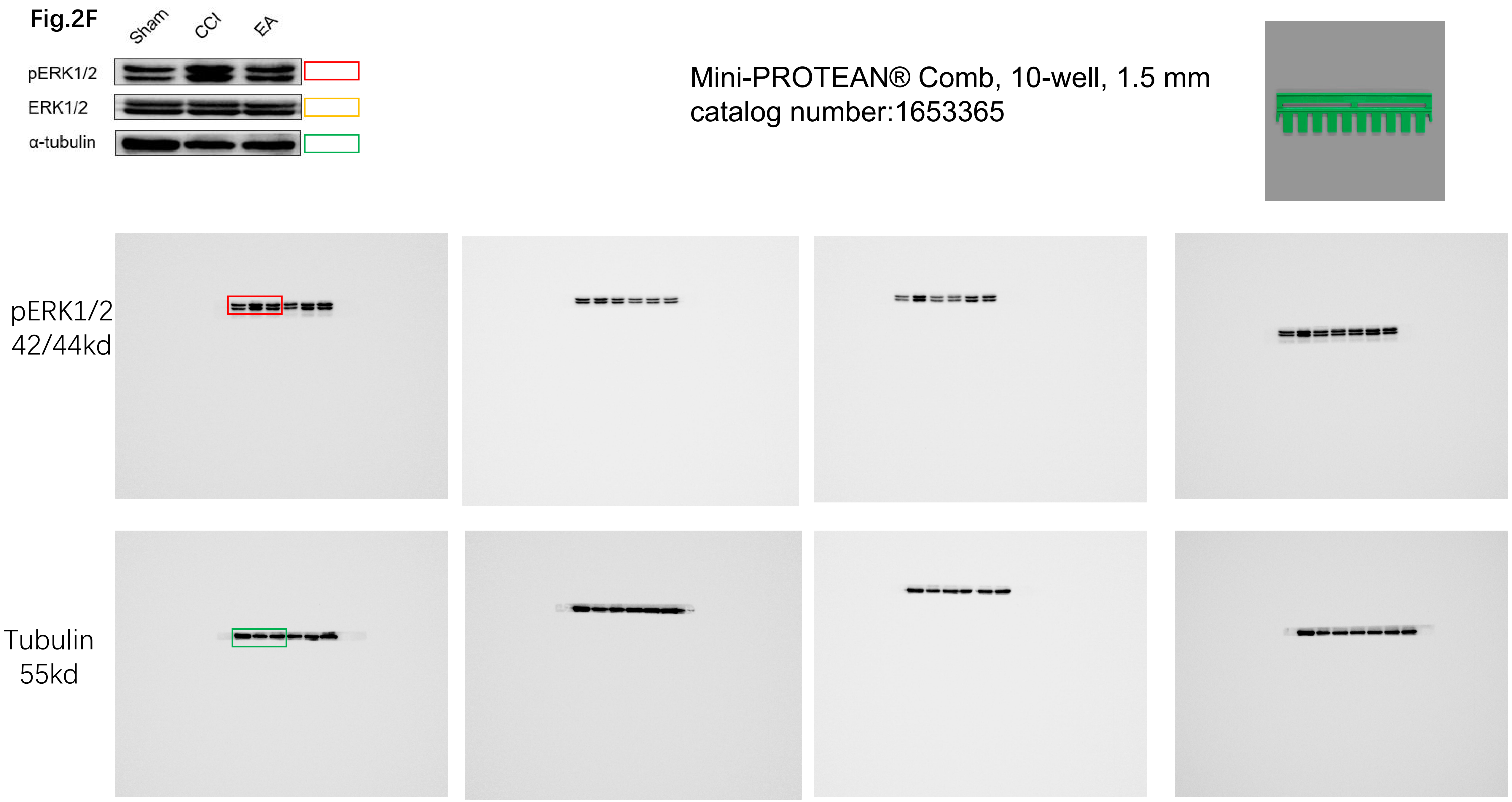
Fig.2F
Sham
CCI
EA
pERK1/2
ERK1/2
α-tubulin
Mini-PROTEAN® Comb, 10-well, 1.5 mm
catalog number:1653365
pERK1/2
42/44kd
Tubulin
55kd

Fig.2F

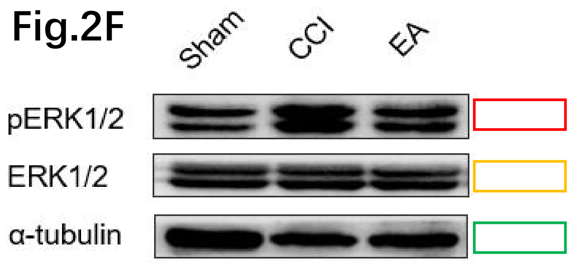

Mini-PROTEAN® Comb, 10-well, 1.5 mm
catalog number:1653365

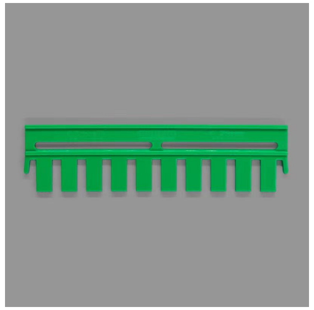

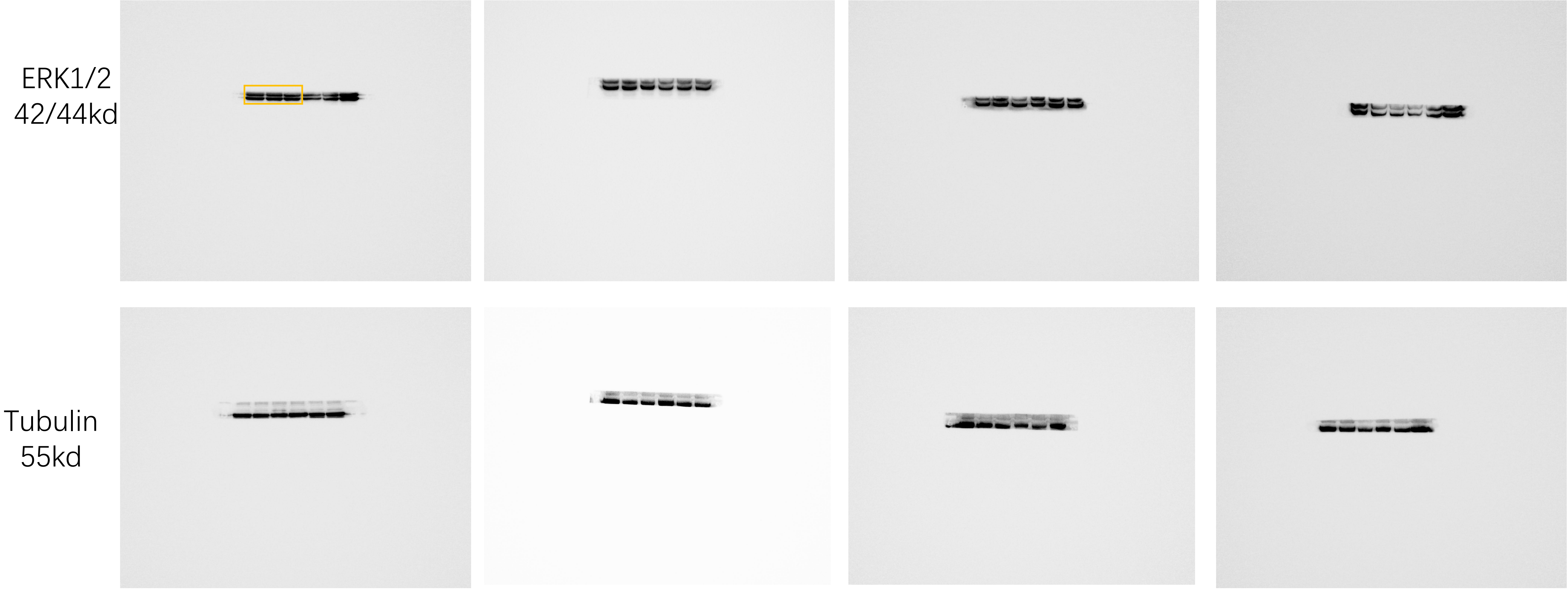

Fig.4E

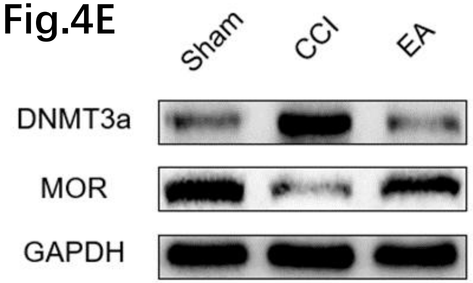

Mini-PROTEAN® Comb, 10-well, 1.5 mm
catalog number:1653365

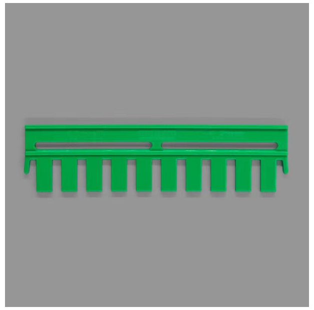

DNMT3a
103kd

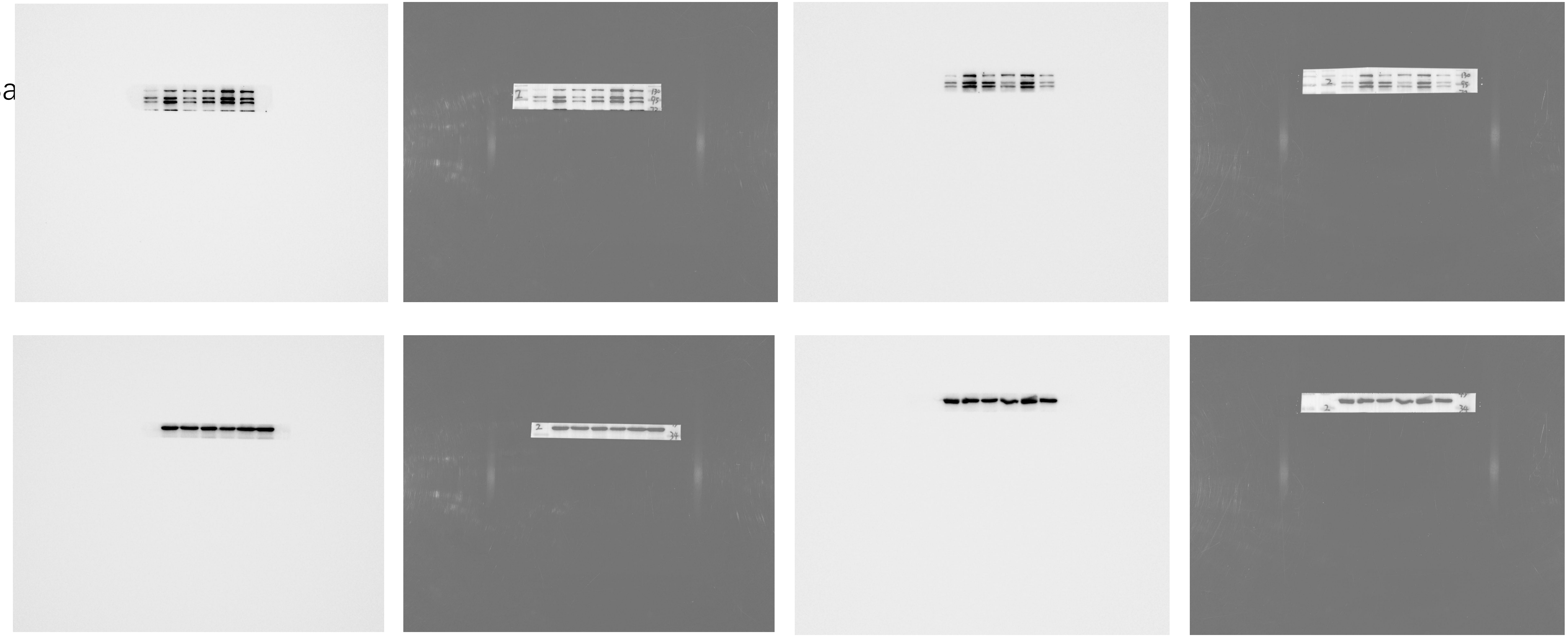

GAPDH
36kd

Fig.4E

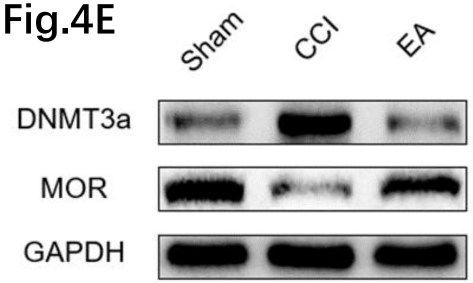

Mini-PROTEAN® Comb, 10-well, 1.5 mm
catalog number:1653365

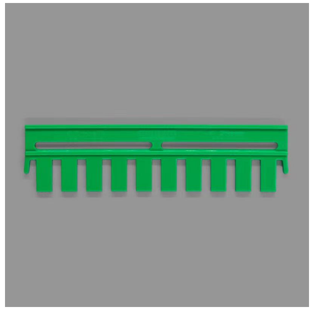

DNMT3a
103kd

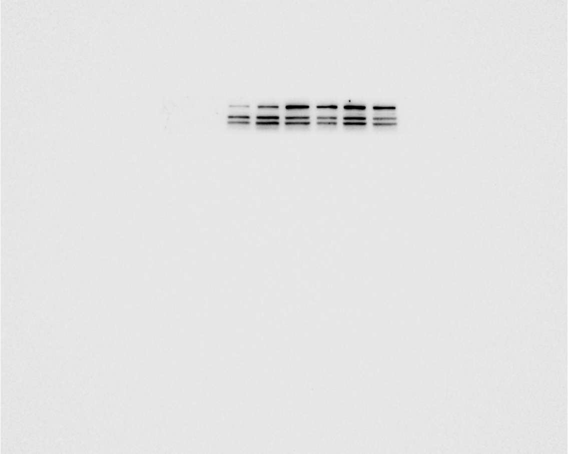

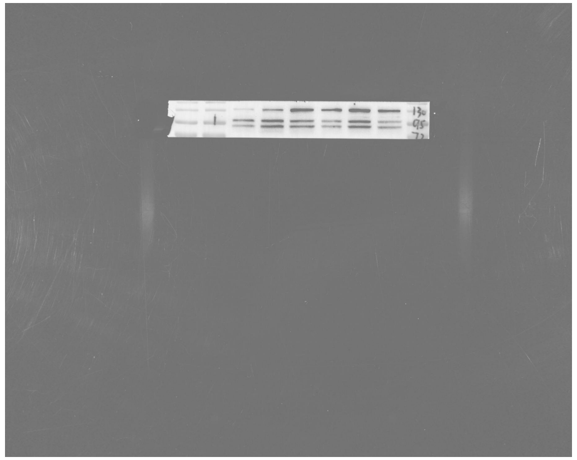

GAPDH
36kd

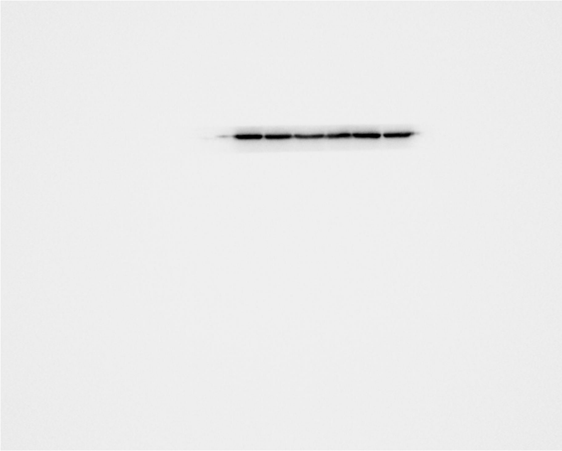

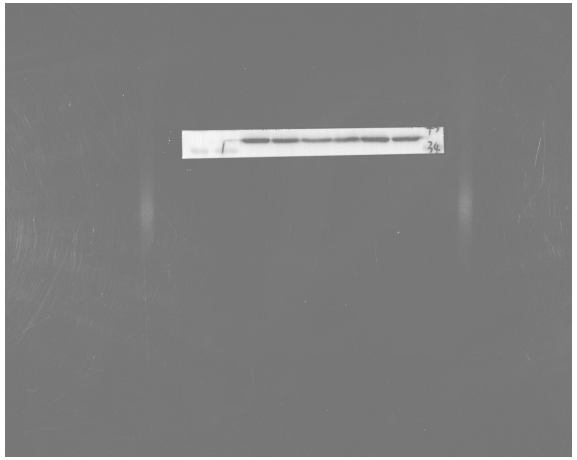

Fig.4E

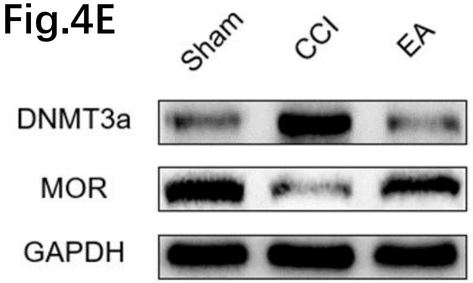

MOR
55kd

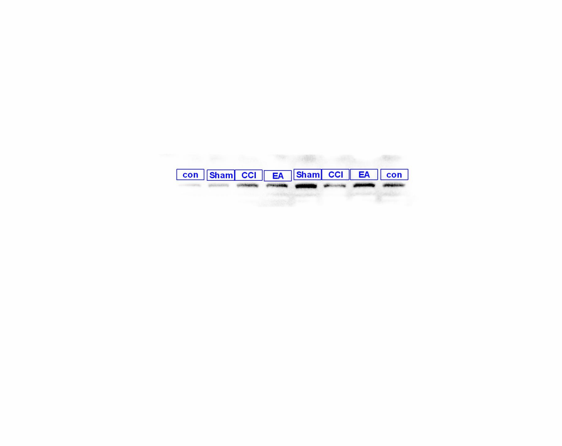

Mini-PROTEAN® Comb, 10-well, 1.5 mm
catalog number:1653365

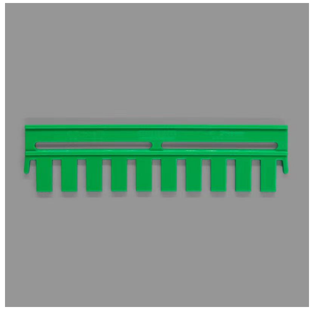

GAPDH
36kd

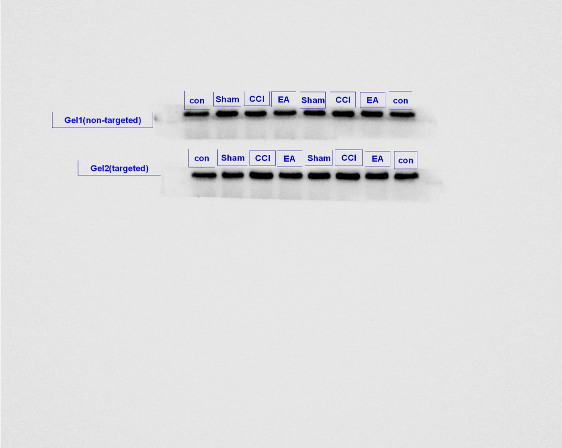

GAPDH in
Bright Field

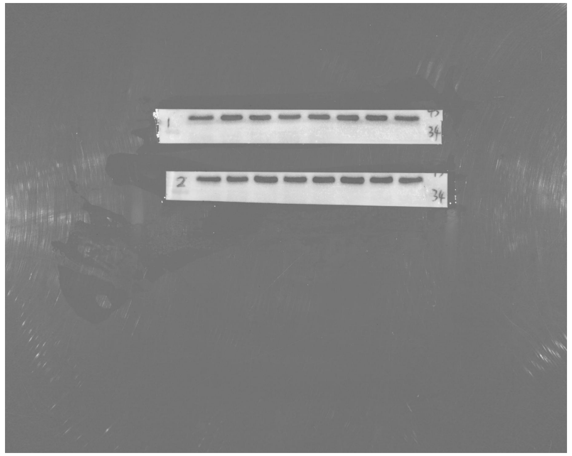

Fig.4E

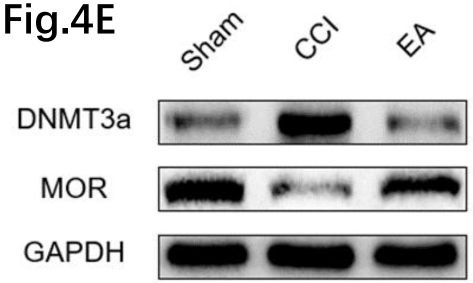

Mini-PROTEAN® Comb, 10-well, 1.5 mm
catalog number:1653365

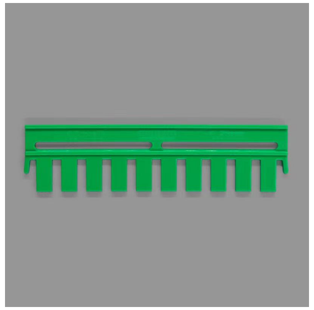

MOR
55kd

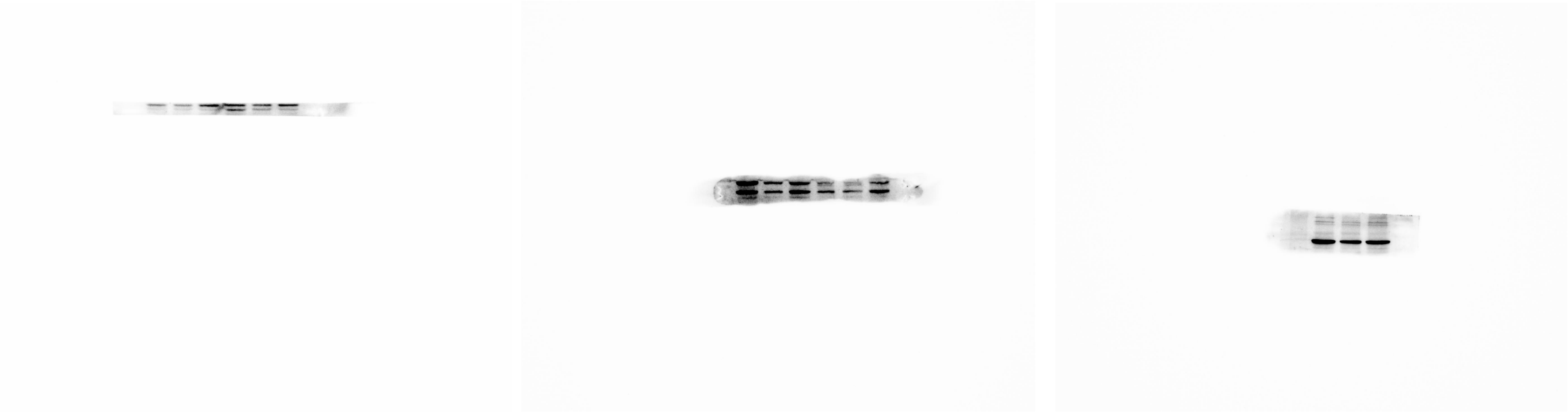

GAPDH
36kd

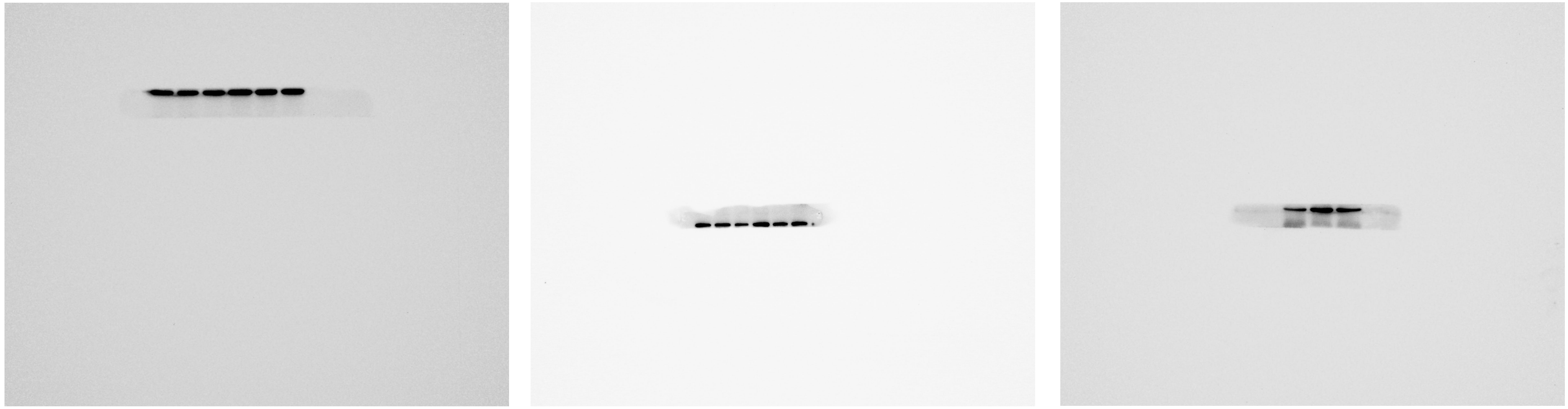

Fig.5c

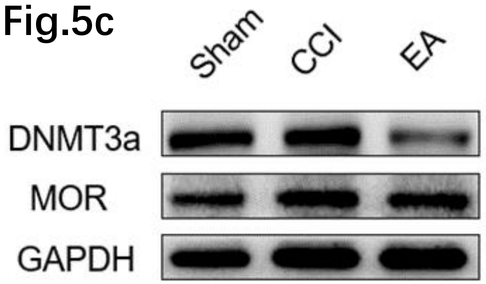

Mini-PROTEAN® Comb, 10-well, 1.5 mm
catalog number:1653365

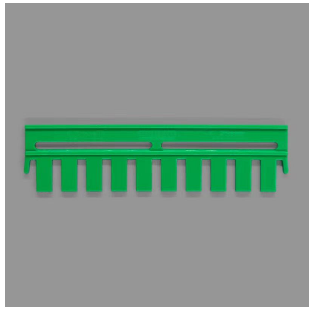

DNMT3a
103kd

GAPDH
36kd

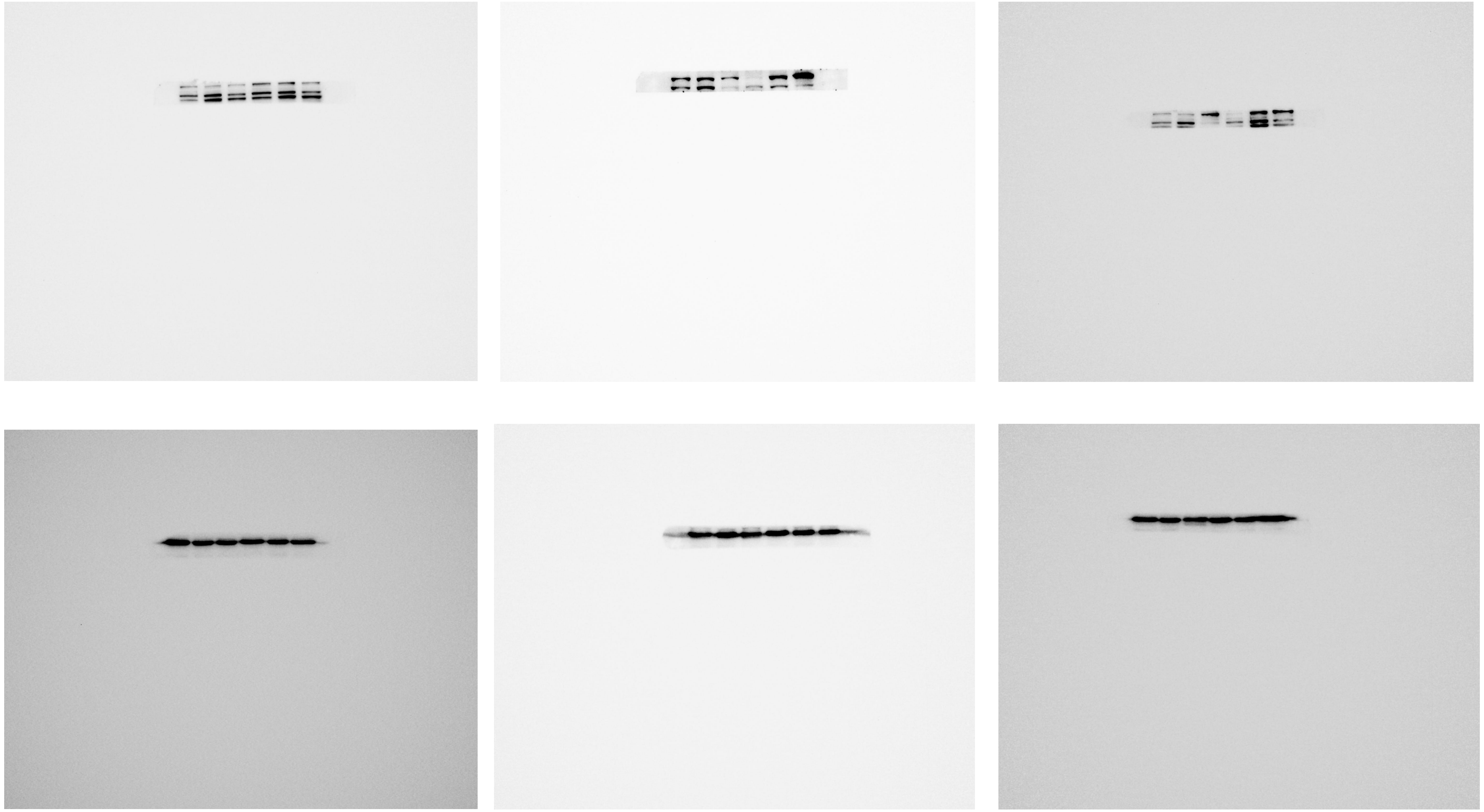

Fig.5c

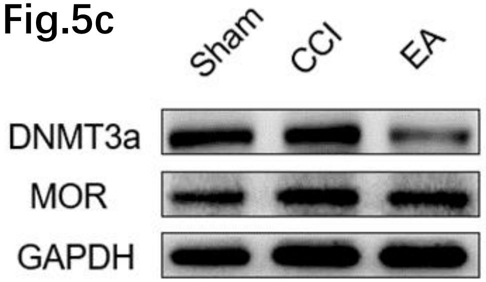

Mini-PROTEAN® Comb, 10-well, 1.5 mm
catalog number:1653365

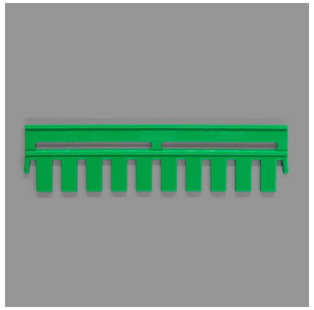

MOR
55kd

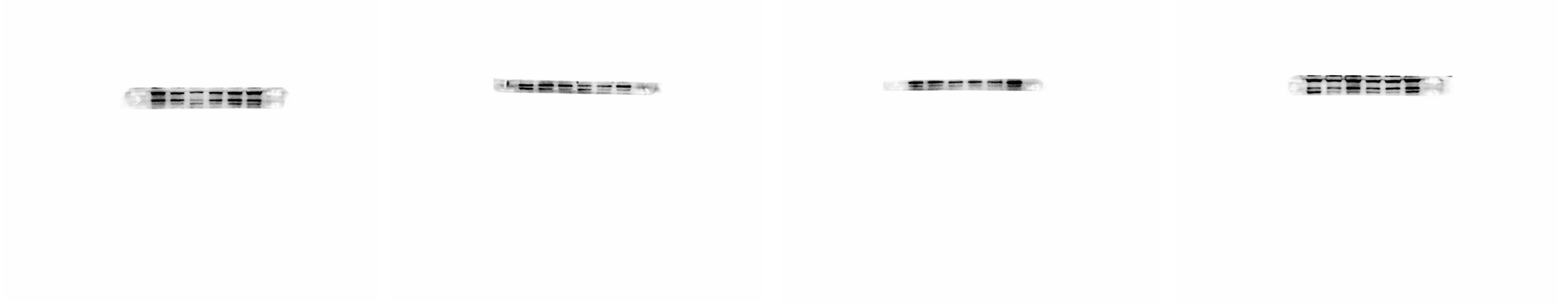

GAPDH
36kd

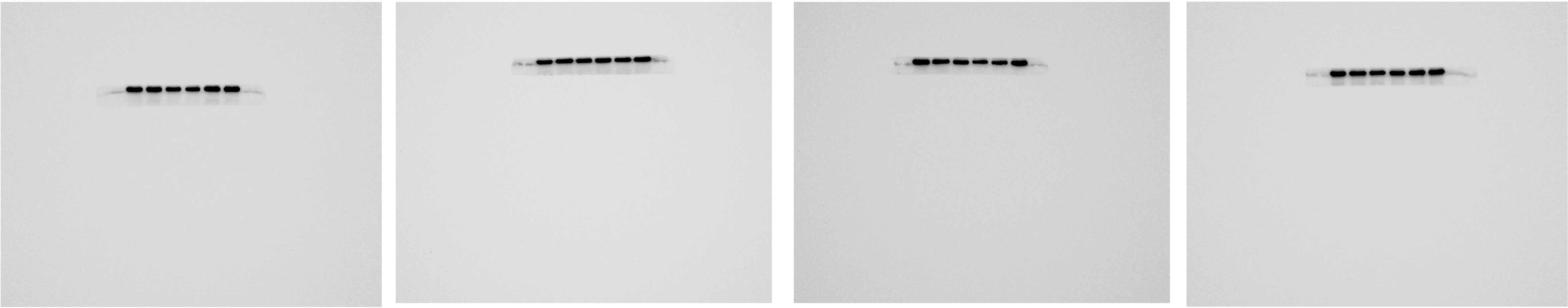

Fig.6D

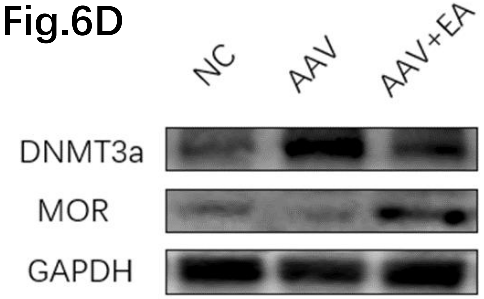

DNMT3a
103kd

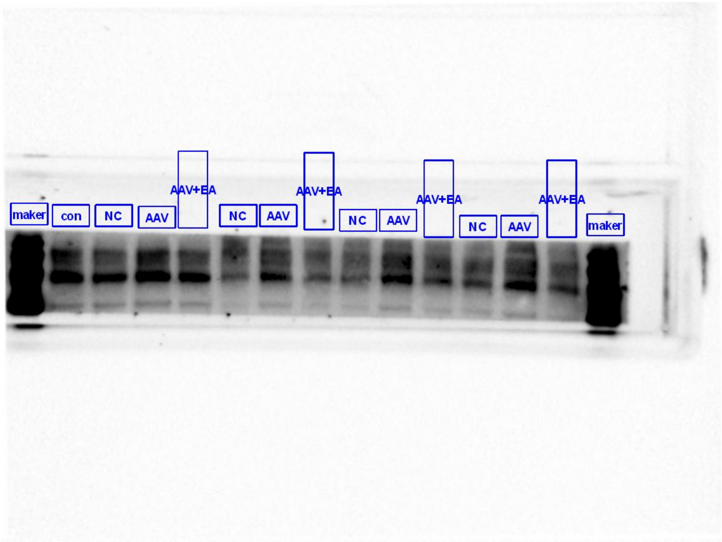

MOR
55kd

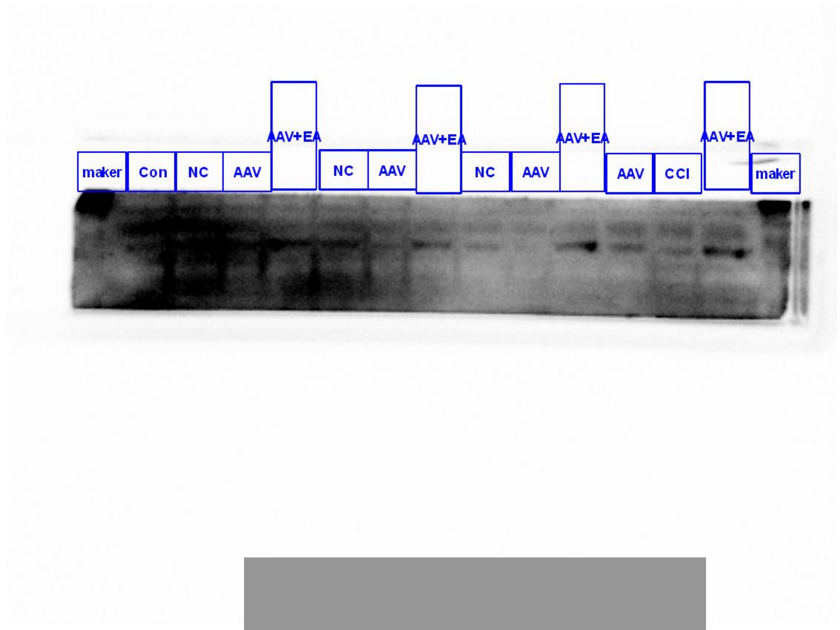

GAPDH
36kd

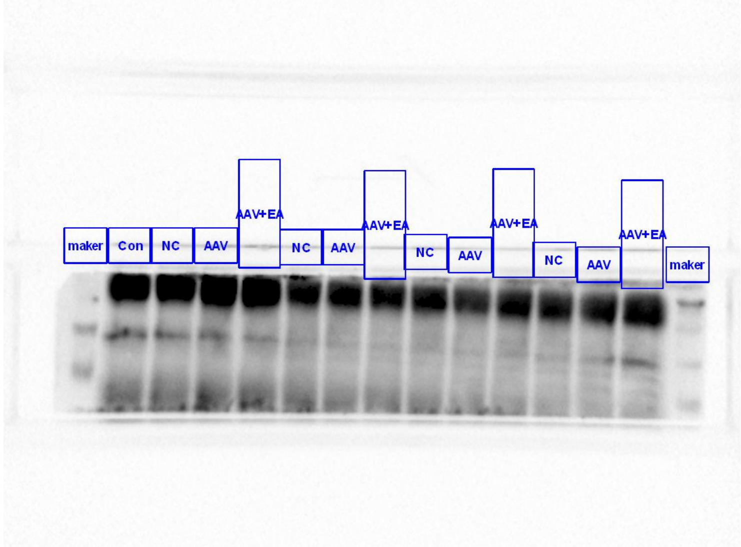

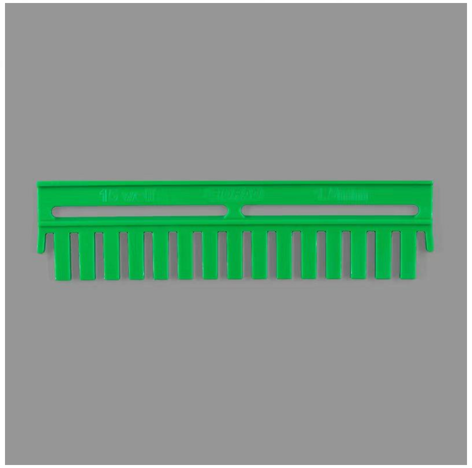

Mini-PROTEAN®Combs, 15-well, 1.5 mm
catalog number:1653366
