# Supplementary material for: Electroacupuncture alleviates pain-like behaviors through modulating DNMT3a/MOR signaling pathway in CCI rats
Source: PLoS One. 2025 Oct 16;20(10):e0334695. doi: 10.1371/journal.pone.0334695 (PMC12530600; doi:10.1371/journal.pone.0334695)
Supplement: Attachment — Submitted filename: Response to Reviewers.docx [file pone.0334695.s004.docx]

**PONE-D-25-32780**

**Response to Reviewers**

Dear Editor and Reviewers:

Thank you for giving us the opportunity to submit a revised draft of the manuscript " Electroacupuncture Alleviates Pain-Like Behaviors Through Modulating DNMT3a/MOR Signaling Pathway in CCI Rats" for publication in the PLOS ONE. We appreciate the time and effort that you and the reviewers dedicated to providing feedback on our manuscript and are grateful for the insightful comments on and valuable improvements to our paper. We have incorporated most of the suggestions made by the reviewers. Those changes are highlighted within the manuscript. Please see below, in blue, for a point-by-point response to the reviewers' comments and concerns.

**Reviewers’ comments to the Authors:**

**Reviewer #1**

**Reviewers’ comment 1:** In the Abstract, the description of the duration of EA treatment is somewhat ambiguous, and the wording should be revised to enhance readability. Also, the full name should be indicated when an acronym appears for the first time.

**Author response:** Added, thanks.

**Modified text:** EA treatment was performed at acupoints Zusanli (ST36) and Yanglingquan (GB34) after CCI 1 week and after AAV dorsal root ganglion (DRG) injection 3 weeks. EA was performed 30 minutes per day for 7 days.

**Reviewers’ comment 2:** Add explanations of the other detected indicators (Fos, ERK1/2, pERK1/2, CD11b, Iba1, and GFAP) in the Introduction section. This will help readers understand the relevance of these indicators to neuropathic pain and the study's mechanistic framework, thereby enhancing the logical coherence of the introduction.

**Author response:** Added, thanks.

**Modified text:** The impact of DNMT3a/MOR on neuropathic pain in CCI model rats was comprehensively evaluated through the observation of changes in pain behavior, pain-related factors (Fos, ERK1/2, and pERK1/2), and glial cell activation (CD11b, Iba1, and GFAP), thereby shedding new light on the potential therapeutic implications of EA in the management of neuropathic pain.

**Reviewer #2**

**Reviewers’ comment 1:** In Figure 2, the authors examined the dynamic changes of Fos mRNA in DRG and SCDH under the CCI model, providing molecular-level evidence for activation of the pain pathway. Notably, they found that transcriptional levels were significantly increased on the ipsilateral side in the CCI group and that electroacupuncture intervention could inhibit this effect, which is a valuable finding. However, relying solely on Fos mRNA levels as evidence of neuronal activation has clear limitations:

① mRNA elevation does not necessarily translate into increased functional c-Fos protein expression, which is the widely accepted marker for neuronal activation.

② The current detection method cannot localize the specific activated cell types (neurons vs. glial cells) or spatial distribution (e.g., specific laminae of the spinal dorsal horn).

Therefore, I strongly recommend that the authors add a double immunofluorescence co-labeling experiment (e.g., c-Fos/NeuN) to confirm the inhibitory effect of electroacupuncture on pain-related neuronal activation, and to specify its location and quantity. If experimental conditions do not allow this, please address in the discussion section:

① clearly state that the conclusions are based only on transcriptional data;

② interpret the results cautiously (e.g., “Increased Fos mRNA suggests possible neuronal activation, which needs further protein validation”);

③ explicitly acknowledge the limitation of not detecting protein expression and cell-type localization.

**Author response:** Thanks. We have accepted the reviewer's suggestions and clearly stated the limitations of this study in the discussion section of the manuscript, including the lack of detection of Fos protein and unclear localization of cell types.

**Modified text:** In the present study, mechanical and thermal nociceptive behavioral tests demonstrated that EA at ST36 and GB34 significantly improved pain sensitivity in rats with CCI of the sciatic nerve. RT-qPCR, western blot, and immunofluorescence techniques were employed to identify changes in Fos, ERK1/2, pERK1/2, lba1, CD11b, GFAP, glial cell activation, DNMT3a, and MOR in the SCDH and DRG. Among them, increased Fos mRNA suggests possible neuronal activation, which needs further protein validationthe.

Therefore, future studies should consider adopting more real-time and dynamic detection methods, such as in vivo imaging, to more accurately assess the efficacy of EA treatment. Similarly, the current detection method cannot localize the specific activated cell types (neurons vs. glial cells) or spatial distribution (e.g., specific laminae of the spinal dorsal horn).

**Reviewers’ comment 2:** The fluorescence images in Figure 3C and Figure 4H are not sufficiently clear. Please provide higher-resolution or clearer images to ensure accurate representation of the results.

**Author response:** Thanks. We have replaced the unclear images pointed out by the reviewer, and the clarity of the replaced images meets the publication requirements.

**Reviewers’ comment 3:** In the mechanistic diagram (Figure 7), the authors propose that CCI can induce upregulation of DNMT3a expression in DRG, along with increased transcription of Oprm1 and Kcna2. However, no experimental data or figures supporting these two targets are provided in the manuscript. Please supplement with relevant data or figures. Additionally, the manuscript mentions a “cascade reaction,” but this should be substantiated experimentally; otherwise, the term should be revised or used more cautiously.

**Author response:** Thanks.

Based on the reviewer's suggestions, we have adjusted the content of the mechanism diagram and removed the targets that were not experimentally validated in this study.

We have removed the inaccurate description of the term 'cascade reaction' mentioned in the article.

**Modified text:**

**Reviewers’ comment 4:** The discussion section is somewhat lengthy and parts of it lack clear structure. It is recommended to restructure this section by adding subheadings or adjusting the logical flow to improve clarity and coherence.

**Author response:** Thanks. According to your suggestion, I have made the modification in the discussion section.

**Modified text:**

**Reviewers’ comment 5:** Please add the full name of DNMT3a in the abstract to help readers better understand the study background.

**Author response:** Added, thanks.

**Modified text:** The expressions of DNA Methyltransferase 3 Alpha (DNMT3a), Mu opioid receptors (MOR), pain-related signals (Fos, ERK1/2, and pERK1/2) and glial cell activity-related factors (CD11b, Iba1, and GFAP) in spinal cord dorsal horn (SCDH) and DRG were detected by western blotting, quantitative polymerase chain reaction reverse transcription (RT-qPCR).

**Reviewers’ comment 6:** Figure 1 lacks a figure number in the manuscript. Please check and correct this. Also, the rat model illustration in Figure 1 appears rather rough; it is recommended to redesign it for better clarity and professionalism. In Figure 2, please add scatter points on the bar charts to indicate the sample size and distribution, which would enhance the transparency of the data.

**Author response:** Thanks.

Based on the reviewer's suggestion, we have checked and confirmed the numbering of Figure 1 in the manuscript.

The rat model in Figure 1 was personally designed and hand drawn by our doctoral team, reflecting our passion for this study and respect for PLOS ONE.

According to the reviewer's suggestion, we have added a scatter plot in Figure 2 to show the sample size and its distribution.

**Reviewers’ comment 7:** Figure 5E and Figure 5F lack serial number labels. Please check and revise accordingly.

**Author response:** Added, Thanks.
